# Injuries in kitesurfing: a retrospective cross-sectional survey on injury patterns based on discipline and skill level, considering time loss and performance reduction

**DOI:** 10.1186/s13102-026-01551-w

**Published:** 2026-01-31

**Authors:** Christoph Offerhaus, Anselm Hohage, Michael Poehlmann, Maurice Balke, Ramona Ritzmann, Christophe Lambert

**Affiliations:** 1 Department of Orthopedic Surgery and Sports Traumatology, Sana Medical Centre, Witten/Herdecke University, Aachener Str. 445-449, Cologne, 50933 Germany; 2 Surfmedizin e.V., Neumuenster, Germany; 3 Witten/Herdecke University, Witten, Germany; 4 Department of Trauma and Orthopedic Surgery, Sports Traumatology, Friedrich-Ebert-Krankenhaus Neumuenster, Neumuenster, Germany; 5 Sportsclinic Cologne, University of Witten/Herdecke, Cologne, Germany; 6 Department of Sport and Sport Science, University of Freiburg, Freiburg, Germany; 7 Innovation Translation Center, AO Foundation, Davos, Switzerland; 8 Orthopädie em Veedel, Praxis für Orthopädie, Unfallchirurgie und Sportmedizin, Cologne, Germany

**Keywords:** Kitesurfing, Kiteboarding, Big air, Foil, Freestyle, Strapless, Sports medicine, Prevention, Sport injury, Athlete, Epidemiology

## Abstract

**Background:**

Despite the rapid growth of kitesurfing, there is a lack of comprehensive scientific research on injuries associated with the sport.

**Hypothesis/purpose:**

To investigate the incidence and patterns of kitesurfing injuries, as well as their impact on time loss and performance reduction across the different subdisciplines and skill levels.

**Methods:**

An online survey among kitesurfers assessed major injuries causing more than three weeks of time loss in kitesurfing, medical treatment, or work absence. Injury frequencies were analysed with reference to discipline, sex, and performance level. Severity was determined by time loss and performance reduction.

**Results:**

3138 athletes reporting 3720 injuries were included into the study. The overall injury rate was 4.8 injuries per 1000 h kitesurfing, ranging from 65/1000 h for beginners to 1.1/1000 h for professionals. Besides cuts and abrasion (23%, *n* = 850), rib injuries (12%, *n* = 448), knee ligament injuries (6%, *n* = 237), foot and ankle fractures (5%, *n* = 193) and ligament injuries (5%, *n* = 174) and concussions (5%, *n* = 171) were most common in kitesurfing. Among the most common injuries, knee ligament injuries were associated with the highest time loss and reduction in performance.

**Conclusion:**

While injury patterns differ by discipline and sex, athlete skill level emerged as the most relevant risk factor, with beginners facing a substantially higher injury risk than advanced or professional kitesurfers. The most severe injuries involved knee ligaments, lower extremity fractures, and rib trauma, resulting in considerable time loss and reduced performance. These findings highlight the need for skill level–adapted and discipline-specific injury prevention strategies.

**Supplementary Information:**

The online version contains supplementary material available at 10.1186/s13102-026-01551-w.

## Introduction

Sports injuries can have serious consequences for both professional and recreational athletes, including post-traumatic degenerative joint changes or the inability to continue participating in sport or competition. The best possible treatment, or even better, effective prevention of these injuries, is one of the main tasks of sports medicine.

A comprehensive understanding of epidemiological injury data not only highlights the most common injuries and their outcomes but also reciprocally serves as the cornerstone for developing effective prevention programs to reduce injury risks [1]. For instance, extensive research into the epidemiology of injuries in team sports has enabled the development of effective, sport-specific prevention programmes, significantly reducing the risk of common and severe injuries [2–6]. 

Since its introduction in the 1990s, kitesurfing has rapidly become one of the fastest expanding water sports. Despite its popularity, research on kitesurfing injury patterns remains limited. The few studies available suggest that injury rates are comparable to those of other sports [7–11]. Two prospective studies report 7 and 10,5 injuries per 1000 h of kitesurfing [12, 13]. At the 2018 Youth Olympic Summer Games in Buenos Aires no injury was reported for kitesurfing [14]. 

The first studies on kitesurfing injuries from the early 2000s showed that half or more of the injuries were due to the inability to separate from the kite in critical circumstances [13, 15]. Since those studies, the sport has evolved considerably in terms of technology and these improvements have significantly increased safety. Nevertheless Wegner et al. reported an increasing injury rate in kitesurfing [10]. In addition to technical advancements that have enhanced safety, recent improvements in kite design and materials have enabled athletes to reach higher speeds and perform more extreme manoeuvres - raising the risk of injury even for recreational athletes.

Furthermore, kitesurfing has diversified into various sub-disciplines over the past decade. Each discipline likely is associated with distinct injury patterns and risks. These sub-disciplines are primarily differentiated by the type of equipment used and the specific manoeuvres performed (freeride, freestyle, park, wave/strapless, big air).

There is no study that sufficiently outlines the aforementioned evolution in kitesurfing and provides a large enough sample size to draw valid conclusions from the described observations. The purpose of our study was to describe the injury rates and patterns in kitesurfing, and to examine the consequences of these injuries with respect to time loss from sport and reduction in sporting performance across the respective disciplines and performance levels.

## Materials and methods

### Study design

In a retrospective study, kitesurfing-related injuries were evaluated through an online survey available in five languages (English, German, French, Spanish, Portuguese). The questionnaire was developed by two sports medicine doctors with kitesurfing experience. Before its release, it was reviewed by five professional kitesurfers and three sports medicine doctors. The survey was designed to only capture major injuries, as these are most relevant to athletes in terms of time loss, functional impairment, and impact on sporting performance. To ensure consistency with established injury epidemiology, commonly used definitions of major injury were applied: injuries were classified as major if they resulted in a time loss of more than three weeks, as defined by Olsen et al., or if they required medical treatment or caused absence from professional duties, as described by Hägglund et al. [6, 16]

All injuries were self-reported, and no clinical verification was required. The questionnaire is provided in the *Supplementary Materials*.

Institutional ethics committee approval was obtained prior to the start of this study (University Witten/Herdecke S-108/2023). All participants or legal representatives provided informed consent to participate in the study, acknowledged the data protection information, and agreed to the anonymous storage and processing of their data, as well as the publication of the results in summarized form.

### Data collection

The final questionnaire consisted of 426 items and was administered as an online survey using the professional platform LimeSurvey. The survey was open from December 2023 to August 2024 and was distributed via Instagram accounts, kitesurfing-related Facebook groups, and mailing lists. The survey comprised three sections.

The first section included questions on the athletes’ demographics, such as age, sex, height, weight, and nationality.

In the second section, participants were asked about their training frequency and intensity, years of experience, and the disciplines they regularly practiced. From this, the total time each participant spent kitesurfing was estimated. Additionally, athletes were asked to classify themselves into one of five performance levels - beginner (kite school, supervised kiting), apprentice (learning independently), intermediate (simple jumps and tricks while hooked), advanced (difficult/high jumps or unhooked), or professional (competing and/or making a living from kitesurfing).

In the third section, participants were asked to report injuries that met the previously mentioned criteria. If an injury was reported, the athlete was asked to provide details about the injury type. For each injury, kite discipline, duration of time loss, performance level upon returning to the sport, and the skill level at the time of the injury were reported. Athletes were asked to report all relevant injuries they had sustained during their careers, based on the previously defined criteria. The reduction in sporting performance after returning to the sport was similarly assessed, with athletes asked to classify themselves into one of three levels: (1) same level, (2) reduced level or (3) had to stop kitesurfing.

### Study population

Any kitesurfer aged 14 and above was eligible to participate, regardless of discipline or skill level. The exclusion criterion was an incomplete questionnaire. Incompleteness was defined as any missing information. Likewise, injuries that were not the focus of the study (e.g., injuries from snowkiting/landboarding) were excluded.

A total of 4408 individuals from 103 countries participated in the survey; 72% identified as male, 27.9% as female, and 0.1% as diverse. Data from 3138 athletes with an average practice period of kitesurfing for seven years met the inclusion criteria and were available for the final analysis. 6.8 million hours of kitesurfing were recorded, of which 5.6 million hours were included in the final analysis.

### Statistical analysis

Primarily, the study cohort was described and analysed on a participant level with regard to demographic characteristics. However, since an individual athlete may sustain multiple injuries during their career, potentially at different skill levels and within different subdisciplines, injuries were subsequently treated as separate analytical units. Accordingly, for the detailed description and analysis of injury characteristics, each reported injury was considered an individual variable and analysed independently of the participant-level data.

Statistical analysis was conducted using SPSS (v.28, SPSS Inc., Chicago, Illinois, USA). Descriptive statistics, including mean ± standard deviation, were calculated for all recorded variables. To illustrate the most common injury types, specific injuries are presented in absolute numbers and as percentages for all athletes, categorized by discipline, sex, and performance level. For clarity, some injuries were grouped based on anatomical region and clinical relevance. For example, rib injuries comprised fractures, contusions, cartilage injuries, and rib dislocations, while knee ligament injuries included ACL, PCL, MCL, and LCL injuries.

To assess differences between disciplines, sex, and sport categories (competitive vs. recreational), risk relationships were analysed using the χ² test and odds ratios (OR). The OR was calculated for each risk factor, with 95% confidence intervals (CIs) constructed.

Time loss intervals and reductions in sporting performance levels after returning to sport for specific injury types are presented as percentages. Differences in these intervals between disciplines, sex, and sport categories were analysed using the Mann–Whitney U test. A p-value of less than 0.05 was considered statistically significant.

### Equity, diversity and inclusion statement

The online survey was open to all kitesurfers regardless of gender, race/ethnicity, or socioeconomic background. The author team comprised one woman and five men from diverse professional backgrounds (medicine, sports science), representing both junior and senior researchers.

## Results

**Fig. 1:**
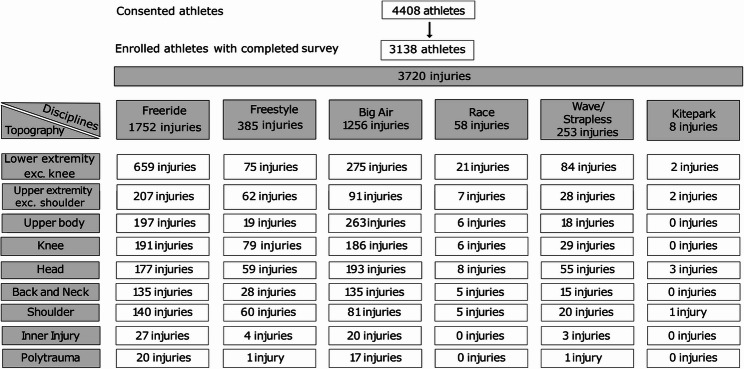
Flow chart diagram reflecting consented and enrolled athletes. Numeric of injuries are illustrated with reference to the body topography (vertical) and disciplines (horizontal). exc. exclusive

Overall, the 3138 included athletes reported 3720 injuries. A proportion of 61.2% (*n* = 1919) of all athletes reported one or more major injuries according to the above-mentioned definition of injury. The number of athletes included in the study, the disciplines and the reported injuries are illustrated in Fig. 1.

### Injury rates

The overall injury rate in kitesurfing was 4.8 ± 34.7 injuries per 1000 h of kitesurfing and did not differ significantly between male (4.0 ± 31.7) and female (6.8 ± 41.2) riders. Beginners experienced the highest injury rates, reporting 65.0 ± 214.5 injuries per 1000 h of kitesurfing. As skill level increased, the injury rate decreased significantly (*p* < 0.001), with 9.7 ± 31.8 among Apprentices, 3.3 ± 7.2 among Intermediates, 1.9 ± 3.0 among advanced riders and 1.1 ± 1.5 among professional athletes.

Kitepark (12.5 ± 66.5 injuries per 1000 h of kitesurfing) was the discipline with the highest prevalence of injuries, followed by Freeride (5.4 ± 38.7). Race (2.7 ± 8.2), Big Air (2.6 ± 6.0), Freestyle (2.1 ± 4.9) and Wave/Strapless (2.1 ± 4.8) showed comparable rates of injuries. (n.s.)

### Injury topography

Across all disciplines and skill levels, the most commonly injured body regions were the lower (43.2%, *n* = 1,607) and upper (18.9% *n* = 704) extremities, specifically knee (13.2%, *n* = 491) and shoulder (8.3% *n* = 307) injuries. Furthermore, athletes reported high rates of upper body (13.5%, *n* = 503) and head injuries (13.3%, *n* = 495). 1% (*n* = 39) of injuries were a polytrauma with need of intensive care in 50% of cases. Compare Fig. 1.

**Fig. 2:**
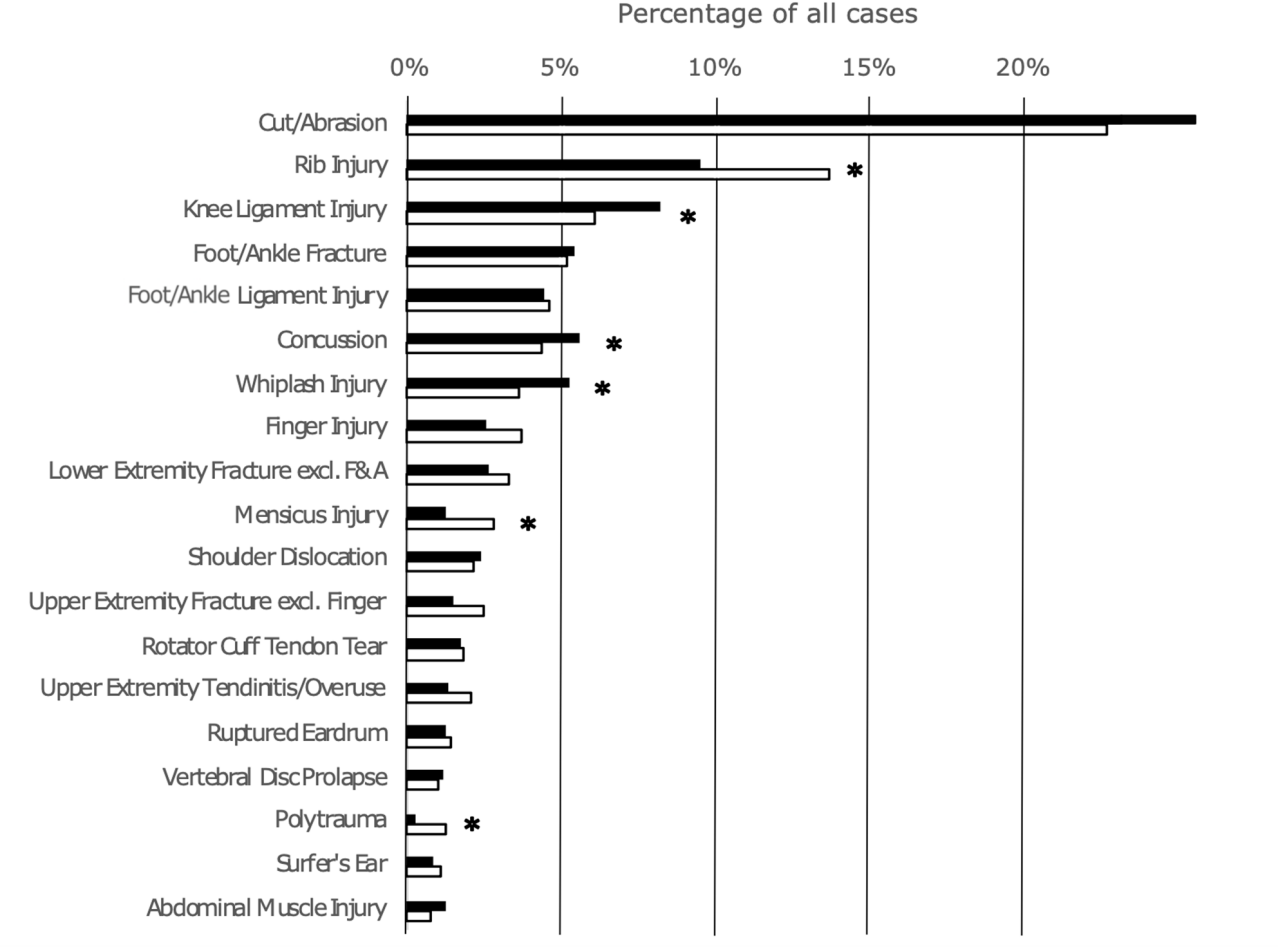
Most common injuries in kite surfing among all disciplines hierarchically clustered from top to bottom and illustrated as percentages of total injury rates for female (black) and male (white) athletes. * Indicates Significant difference. F&A Foot and Ankle, excl. exclusive

The most common injuries in the overall cohort were cuts or abrasions (22.9%, *n* = 850) and rib injuries (12.0%, *n* = 448), followed by knee ligament injuries (6.4%, *n* = 237) foot and ankle fractures (5.2%, *n* = 193) and foot and ankle ligament injuries (4.7%, *n* = 174), concussions (4.6%, *n* = 171), and whiplash injuries (4.1%, *n* = 153). A detailed breakdown of the most common injuries can be found in Fig. 2.

### Kitesurfing disciplines and injuries

Regarding kitesurfing disciplines, 72.3% of participants engaged in Freeride (76.3% females, 70.8% males, *p* < 0.001), 45.5% in Big Air (26.5% females, 52.9% males, *p* < 0.001), 26.1% in Wave/Strapless (24.0% females, 27.0% males, n.s.), 15.3% in Freestyle (14.2% females, 15.7% males, n.s.), 4.0% in Race (3.8% females, 4.0% males, n.s.), and 1.2% in Kitepark (0.6% females, 1.5% males, n.s.).

**Fig. 3:**
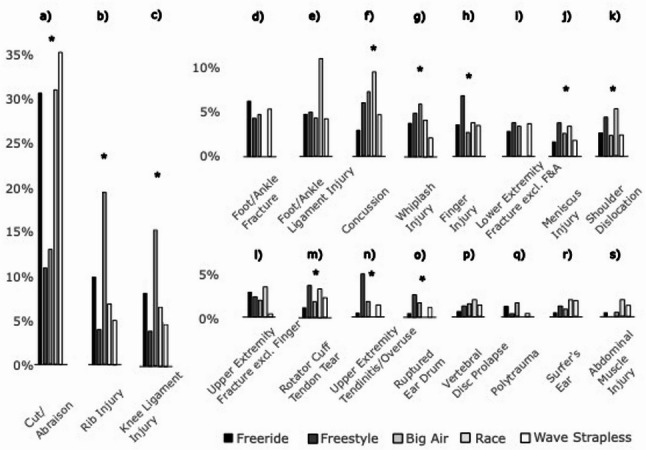
Injury pattern with reference to the kite discipline Freeride, Freestyle, Big Air, Race and Wave/Strapless. The Percentages on the y-axis refer to the proportion of injury type per kite discipline with the sum off all being 100%. * Indicates Significant difference. F&A Foot and Ankle, excl. exclusive

Injury patterns varied significantly across kitesurfing disciplines. A detailed overview of injuries by discipline can be found in Fig. 3. The largest cohort participated in Freeride, with the most common injuries being cuts and abrasions, accounting for 30.7% (*n* = 538) of injury cases, followed by rib injuries (9.7%, *n* = 169) and foot and ankle fractures (6.2%, *n* = 108). Cuts were prevalent across all disciplines, particularly in Wave/Strapless (35.2%, *n* = 89) and Race (31.0%, *n* = 18). Athletes engaging in Big Air predominantly sustained rib injuries (19.3%, *n* = 242), concussions (7.2%, *n* = 90), and knee ligament injuries (6.9%, *n* = 87). In Freestyle, knee ligament injuries (10.1%, *n* = 39), finger injuries (6.8%, *n* = 26), and overuse injuries/tendinitis of the upper extremities (5.2%, *n* = 20) were most common. In addition to the aforementioned high incidence of cuts and abrasions, athletes in the Race discipline primarily suffered ligament injuries to the foot and ankle (10.3%, *n* = 6) or concussions (8.6%, *n* = 5), whereas those in Wave/Strapless were most affected by knee ligament injuries (5.9%, *n* = 15) or foot and ankle fractures (5.5%, *n* = 14). Most common injuries in Kite Park were cuts and concussions; however, due to the small sample size (*n* = 8 injuries) in this cohort, they are not further analysed in this interdisciplinary comparison. While upper extremity injuries were generally rare, shoulder dislocations represented 5.2% of all injuries in Race and 4.7% in Freestyle.

### Skill level and injuries

Overall, 83.6% of athletes who reached a professional level in kitesurfing reported having sustained at least one major injury at any point during their kitesurfing career, compared with 60.3% of recreational-level athletes.

When injuries were analyzed on an injury-based level according to the activity during which they occurred, professional kitesurfing was associated with significantly higher rates of knee ligament injuries, concussions, and shoulder dislocations. In contrast, injuries sustained during recreational kitesurfing more frequently consisted of cuts and abrasions as well as foot and ankle fractures Table 1.

**Table 1:** Injury risk depending on sex and performance level

	Male vs. female				Professional vs. Recreational			
Injury	OR	95% CI lower	95% CI upper	p-value	OR	95% CI lower	95% CI upper	p-value
Cut/Abrasion	0.85	0.72	1.02	0.074	**0.55**	0.38	0.79	**0.001**
Rib injury	**1.46**	1.14	1.87	**0.002**	0.66	0.42	1.05	0.076
Knee ligament injury	**0.72**	0.55	0.96	**0.025**	**1.90**	1.24	2.91	**0.003**
Foot/ankle fracture	0.97	0.69	1.35	0.837	**0.42**	0.19	0.97	**0.036**
Foot/ankle ligament injury	1.07	0.75	1.54	0.705	1.22	0.69	2.13	0.497
Concussion	**0.69**	0.49	0.98	**0.035**	**2.01**	1.22	3.32	**0.005**
Whiplash injury	**0.62**	0.44	0.89	**0.009**	1.03	0.53	1.98	0.937
Finger injury	1.53	0.95	2.47	0.077	1.31	0.67	2.54	0.424
Lower extremity fracture excl. F&A	1.29	0.82	2.04	0.265	1.76	0.97	3.19	0.058
Meniscus injury	**2.19**	1.19	4.04	**0.010**	1.06	0.46	2.46	0.892
Shoulder dislocation	0.79	0.48	1.29	0.343	**2.23**	1.16	4.27	**0.013**
Upper extremity fracture excl. fingers	1.53	0.85	2.74	0.151	0.75	0.27	2.08	0.581
Rotator cuff tendon tear	1.09	0.60	1.99	0.778	1.74	0.78	3.86	0.170
Upper extremity tendinitis/overuse	1.44	0.78	2.64	0.240	1.67	0.71	3.94	0.237
Ruptured eardrum	0.99	0.50	1.98	0.987	2.03	0.78	5.24	0.137
Vertebral disc prolapse	0.90	0.45	1.81	0.774	0.33	0.04	2.40	0.248
Polytrauma	**4.02**	1.23	13.08	**0.012**	1.52	0.54	4.31	0.428
Surfer’s ear	1.38	0.57	3.39	0.474	**4.50**	1.77	11.43	**0.001**
Abdominal muscle injury	0.60	0.30	1.23	0.159	1.66	0.38	7.26	0.497

Significant results are shown in bold

*OR* Odds ratio, *CI* Confidence interval

### Sex and injuries

Compared to their female counterparts, male athletes reported significantly higher rates of rib and meniscus injuries. The risk of polytrauma was four times higher for male athletes. Female athletes, showed a higher risk of concussions, whiplash injuries, and knee ligament injuries Table 1.

### Time loss

**Fig. 4:**
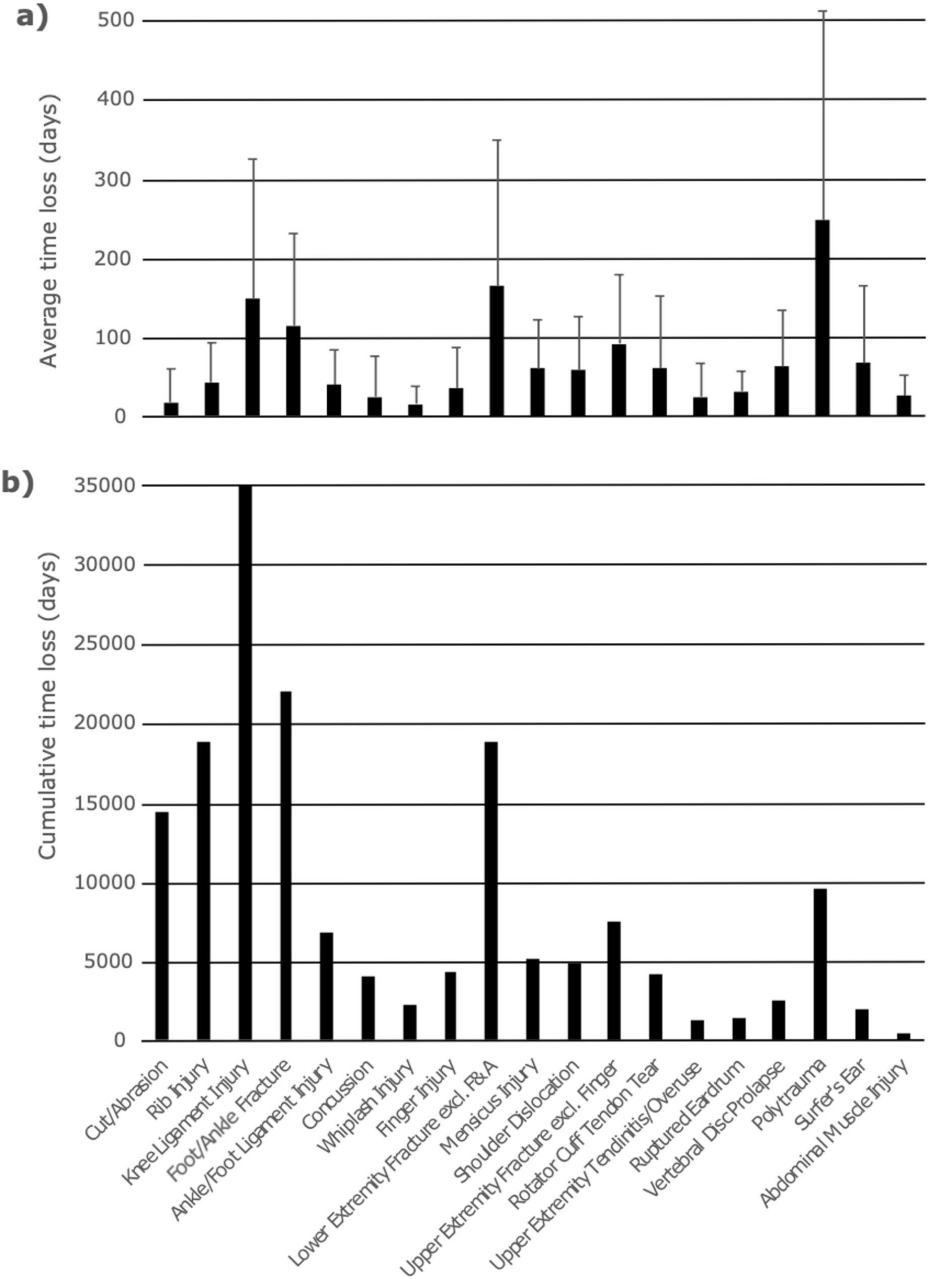
Time loss due to injury: (**a**) plot of average time loss on the y-axis with reference to the recorded injuries on the x-axis and (**b**) cumulated time loss as the product of injury x time loss on the y-axis among all participants and injuries per injury cluster on the x-axis. F&A Foot and Ankle, excl. exclusive

Across all performance levels, the longest time loss was observed following polytrauma (247 ± 264 days), exceeding eight months on average. However, this injury was reported by only 1.1% (*n* = 39) of participants. An average time loss of three to six months was reported for knee ligament injuries (148 ± 176 days) and lower extremity fractures (163 ± 185 days), including foot and ankle fractures (114 ± 116 days). An average time loss of six weeks to three months was observed for fractures of the upper extremities, excluding finger fractures (91 ± 87 days), vertebral disc prolapses (62 ± 71 days), rotator cuff tendon tears (60 ± 91 days), meniscus injuries (59 ± 62 days), shoulder dislocations (58 ± 67 days) and rib injuries (42 ± 50). An average time loss of three to six weeks was reported for foot and ankle ligament injuries (39 ± 45 days), finger injuries (35 ± 51 days), ruptured eardrums (31 ± 25 days), concussions (24 ± 51 days) and tendinitis of the upper extremities (24 ± 42 days). The shortest time loss with less than three weeks, was observed for cuts/abrasions (17 ± 42 days) and whiplash injuries (15 ± 22 days) Fig. 4a.

Cumulative time loss identifies knee ligament injuries (35,076 days), fractures of the lower extremities (18,908 days) - particularly of the foot and ankle (22,002 days) - and rib injuries (18,816 days) as the most relevant in kitesurfing Fig. 4b.

### Return to sports

Among the most common injuries, athletes with knee ligament injuries (64.3%) and lower extremity fractures except foot and ankle (62.6%) showed the lowest likelihood of returning to their pre-injury performance level. Overall, polytrauma (53.9%) and vertebral disc prolapse (61%) were associated with the greatest reduction in performance level; however, their incidence was considerably lower compared to the aforementioned injury types. Following a polytrauma, 5.1% of the participants reported stopping kitesurfing Table 2.

**Table 2:** Reduction in performance level upon return to sport after the most common injuries

		Reduction of Performance level (%)		
Injury Types	N	Same level	Reduced level	Stop Kitesurfing
Cut/Abrasion	849	95.9%	3.8%	0.4%
Rib Injury	444	89.9%	9.9%	0.2%
Knee Ligament Injury	230	64.3%	35.2%	0.5%
Foot/Ankle Fracture	193	74.1%	24.9%	1.0%
Foot/Ankle Ligament Injury	171	86.5%	13.5%	0.0%
Concussion	168	89.9%	8.9%	1.2%
Whiplash Injury	152	89.5%	9.9%	0.7%
Finger Injury	123	91.1%	8.1%	0.8%
Lower Extremity Fracture excl. F&A	115	62.6%	35.7%	1.7%
Mensicus Injury	84	72.6%	27.4%	0.0%
Shoulder Dislocation	85	74.1%	25.9%	0.0%
Upper Extremity Fracture excl. Finger	80	88.8%	10.0%	1.3%
Rotator Cuff Tendon Tear	69	68.1%	31.9%	0.0%
Upper Extremity Tendinitis/Overuse	57	75.4%	22.8%	1.8%
Ruptured Eardrum	46	100.0%	0.0%	0.0%
Vertebral Disc Prolapse	41	61.0%	39.0%	0.0%
Polytrauma	39	53.9%	41.0%	5.1%
Surfer’s Ear	29	79.3%	20.7%	0.0%
Abdominal Muscle Injury	18	94.4%	5.6%	0.0%

Foot and ankle fractures, meniscal injuries, shoulder dislocations, rotator cuff tears, and tendinopathies or overuse injuries of the upper extremity were also associated with a notable reduction in performance, with only 70–75% of athletes returning to their pre-injury performance level. High return-to-performance rates around 90% were observed following rib injuries, foot and ankle ligament injuries, concussions, whiplash injuries, and fractures of the upper extremity Table 2.

Injury-related reduction of performance did not differ significantly between professional and recreational athletes.

## Discussion

This study provides a comprehensive dataset on injuries in kitesurfing, distinguishing injury patterns across the most common kitesurfing sub-disciplines, including all skill levels from beginners to professionals. It is the first study to analyse injury-specific time loss and the associated reduction in athletic performance level in kitesurfing.

### Injury rates

The rate of 4.8 injuries per 1,000 h represents the lowest scientifically documented overall injury rate in kitesurfing, which can be attributed to the fact that only major injuries were recorded. Outcomes are in line with two prospective studies reporting injury rates between 7.5 and 10 injuries per 1000 h of kitesurfing [12, 13] and are comparable to injury rates in common team sports like soccer that range from 4.6 to 9.9 injuries per 1,000 h [17]. The most comparable sport, windsurfing, reports lower injury rates ranging from 0.2 to 5.2 per 1000 h [8, 18, 19]. 

Beginners exhibited the highest injury rates, suggesting that inadequate technical skills, poor judgment, exposure to shallow water during exercise, or unfamiliarity with safety systems and manoeuvres may be critical risk factors at early stages of the sport [7, 8, 12, 13]. Although rates of 65 injuries per 1,000 h of kitesurfing may overestimate the true incidence - since beginners sustaining an injury and subsequently discontinue kiting may disproportionately inflate the rate per 1,000 h - the extremely high injury numbers represents a paramount concern. In a psychological study, Wegner et al. also reported a significantly elevated injury rate of 31 injuries per 1,000 h among beginners compared to advanced kiters [10]. As skill level increased, injury rates decreased significantly, supporting the hypothesis that experience is protective. Interestingly, this contrasts with most other sports, where injury rates increase with advanced expertise [20–22]. In other skill-based outdoor sports such as skiing and snowboarding, for example, both injury rates and injury severity have been shown to rise with increasing skill level [23]. In team and combat sports for instance, the mechanical loads experienced by athletes increase with higher performance levels as opponents become physically stronger and faster, resulting in greater forces and torques during training and contest situations [24]. In contrast, kitesurfing performance is primarily influenced by environmental factors such as wind strength and wave conditions, rather than by direct physical interactions with other athletes.

Advanced disciplines like Race, Freestyle, and Big Air had the lowest injury rates, indicating that skill level may outweigh discipline in injury risk. However, the advanced discipline of Kitepark represents an exception, with the highest injury rate across all disciplines - likely due to obstacle-related collisions.

### Time loss and return to sports

Among the common kitesurfing injuries, knee ligament injuries, followed by fractures of the lower extremities, especially the foot and ankle, were the most severe injuries with the longest time loss and significantly reduced performance after return to sport. Interestingly, and in contrast to most other sports, rib injuries are notably frequent in kitesurfing, and, despite moderate time loss per case, cumulatively relevant. Polytrauma is a rare but devastating injury in kitesurfing.

### Discipline-specific injury profiles

Injury patterns vary significantly across the different kitesurfing sub-disciplines.

Freeride was associated with a high incidence of less severe injuries such as cuts and rib injuries. Nevertheless, athletes in Freeride also sustained a considerable number of foot and ankle fractures most likely due to exposure to shallow water. Crashes in Big Air frequently occur from heights up to 20 m with high incidence of rib injuries, concussions and whiplash injuries. Knee ligament injuries were also common in Big Air but occurred most frequently in Freestyle. Furthermore, Freestyle was the discipline associated with a higher rate of upper extremity injuries, such as shoulder dislocations, finger injuries, and overuse injuries of the upper limb. Athletes in racing suffered the highest rate of ligament injuries to the foot and ankle.

### Sex differences

Outcomes reflect both anatomical and behavioural differences in risk-taking, injury mechanics and muscular control strategies between the sexes which is in line with other sports and emphasizes the need for sex-specific training and prevention protocols [10, 25–30]. 

Female athletes showed significantly higher incidences for concussions and whiplash injuries. This is consistent to previous studies reporting female athletes to be more susceptible to concussions than males, due to anatomical and physiological differences such as reduced neck strength and lower biomechanical thresholds [29–31]. Furthermore, female kitesurfers reported a significantly higher incidence of knee ligament injuries. This finding is consistent with previous research, particularly from team sports and soccer, indicating that women have a two- to eightfold increased risk of anterior cruciate ligament injuries [26, 27]. This elevated risk is attributed to a combination of anatomical and neuromuscular factors, as well as hormonal influences on ligament laxity [25, 27, 28, 32]. The significantly higher risk of polytrauma and rib injuries among male kitesurfers may be associated with a greater tendency towards risk-taking compared to their female counterparts. Recent evidence demonstrated a correlation between higher sensation-seeking scores and increased injury risk in kitesurfing [10]. 

### Limitations

There were two limitations to our study:

Data were collected retrospectively and injuries and their severities were self-reported, which carries a risk of diagnostic misclassification. Previous research in football has shown that only 61% of players were accurate when recalling their exact diagnosis in a twelve months injury history [33]. This recall bias represents a major limitation and may have led to both underreporting and/or overreporting compared to prospective longitudinal designs. However, in the above-mentioned study a 100% recall was shown for the presence or absence of injuries and 80% were able to accurately recall the number of injuries and body regions injured.

Injury rates were calculated through correlating self-estimated sport exposure to injury frequency. Accurately reconstructing exposure hours retrospectively is inherently challenging, and the reported values should therefore be considered approximate estimates rather than precise measurements. A prospective assessment would represent the optimal approach for exposure quantification. However, rather than reporting specific incidences for comparison with other sports, the main goal of the present study was to document injury proportions and injury-specific time losses and sporting performance reductions. Additionally, any recall-related imprecision in exposure estimation would likely have affected all injury categories in a comparable manner.

Additionally, a selection bias cannot be fully excluded. Athletes who sustained severe injuries may have discontinued kitesurfing and, as a consequence, may no longer be reachable through sport-related social media channels used for study recruitment. On the other hand, participants with a history of injury may have been more inclined to respond. This selection bias may have led to both underreporting and/or overreporting.

## Conclusion

This study provides the first large-scale analysis of injury patterns, time loss, and performance impact across different kitesurfing disciplines and skill levels. While discipline-specific and sex-specific injury patterns can be identified, skill level appears to be a more critical risk factor with beginners showing a significantly higher injury risk compared to advanced or professional athletes. Knee ligament injuries, lower extremity fractures, and rib injuries were identified as the most impactful injuries in terms of cumulative time loss and performance reduction. The observed injury patterns in kitesurfing emphasize the importance of implementing prevention strategies tailored to sex and discipline, as well as enhancing experience-based instruction and decision-making guidance during the early stages of progression. Future studies will aim to analyse both the underlying injury mechanisms and the equipment involved in greater detail.

## Supplementary Information

Supplementary Material 1

## Data Availability

The data that support the findings of this study are not openly available but are available from the corresponding author upon reasonable request.
