# Supplementary material for: Injuries in kitesurfing: a retrospective cross-sectional survey on injury patterns based on discipline and skill level, considering time loss and performance reduction
Source: BMC Sports Sci Med Rehabil. 2026 Jan 31;18:114. doi: 10.1186/s13102-026-01551-w (PMC12969894; doi:10.1186/s13102-026-01551-w)
Supplement: Supplementary file 1 — Supplementary Material 1 [file 13102_2026_1551_MOESM1_ESM.pdf]

General Questionnaire

1. **How old are you (years)?**

2. **Are you female, male, or diverse?**

3. **How tall are you (cm)?**

4. **How much do you weigh (kg)?**

5. **Which country do you live in?**

6. **How many years have you been kitesurfing?**

7. **How often do you typically go kitesurfing per month (number of sessions)?**
   (If the number of sessions varies significantly due to the season, vacations, or weather conditions, please calculate/estimate the approximate monthly average)

   How often do you typically go kitesurfing per year (number of sessions)?

8. How many hours do you typically kitesurf per session?
(Calculate/estimate the approximate average here as well)

9. How would you rate your skill level?
Beginner (Kite school, supervised kiting)
Apprentice (learning independently)
Intermediate (simple jumps and tricks hooked)
Advanced (difficult/high jumps or unhooked)
Professional (competitions and/or making a living from kitesurfing)

10. Which disciplines do you practice? (Multiple choices possible)
Freeride (recreational riding, no specific maneuvers or jumps)
Freestyle (unhooked)
Big Air
Race
Wave/Strapless
Kitepark (Obstacles)

11. Do you participate in competitions? (Multiple choices possible)
No
Race
Slalom
Freestyle
Big Air
Wave
Strapless
Kitepark (Obstacles)
Other:

12. Do you earn money with kitesurfing?
No

Yes, part-time
Yes, full-time

If yes, how do you earn money?
Competitions
Sponsorship
Social Media (YouTube/Instagram/TikTok/etc.)
Other

If yes, what is your main source of income?
Competitions
Sponsorship
Social Media (YouTube/Instagram/TikTok/etc.)
Other

13. On which type of water do you typically kitesurf?
Ocean flat water
Ocean waves
Lake
River

14. With which board(s) do you typically kitesurf? Multiple answers possible!
Twintip with foot straps
Twintip with boots
Directional board / Waveboard without foot straps
Directional board / Waveboard with foot straps
Foilboard without foot straps
Foilboard with foot straps
Other:

15. Do you use a safety leash?
No
Yes, attached to the back of the harness
Yes, attached to the side or front of the harness

16. Do you use a board leash?
No
Yes, retractable leash
Yes, spiral/coiled leash
Yes, rigid leash

17. Do you wear protective equipment? (Multiple answers possible)
No
Impact vest
Helmet
Earplugs
Other:

Specific Questionnaire: Injuries in Kitesurfing

We will now ask you some questions about injuries in kitesurfing. We assume that bruises and the occasional scrape are part of the sport. When we ask about injuries, we mean those that resulted in you having to undergo medical treatment, or rendered you unable to kitesurf/train for at least 3 weeks or be unable to work for at least 1 day.

18. Have you ever been injured while kitesurfing?
Yes
No, no injuries that meet the criteria mentioned above (→ Question 44)

19. Which of the following injuries have you sustained while kitesurfing? (Multiple choices possible)
Polytrauma
Concussion
Whiplash (cervical spine sprain)
Internal organ injury
Bone fracture
Ligament tear
Dislocation
Cut/tear injury
Joint sprain
Strain/muscle tear
Bruise

The following is a detailed survey of injuries in various body regions. For any injury you mention, the following details about the injury and downtime will be requested!

a. Where did the injury occur?
Instruction
Recreational
Training
Competition
Video shoot or similar

b. In which discipline did the injury occur?
Freeride (no specific maneuvers or jumps)
Freestyle (unhooked)
Big Air
Race
Wave/Strapless
Kitepark (Obstacles)

c. How did the injury happen?
Kite launch/landing
Flight training (exercises without board) on land
Flight training (exercises without board) in water
Normal riding
Speed riding/racing/slalom
Jump hooked
Jump unhooked
Jump with kiteloop (hooked or unhooked)
Other maneuver

d. Cause of the injury:
Riding error (lost control during normal riding)
Maneuver/trick (intentional maneuver/trick – lost control during execution)
Environmental factors (lost control due to external factors - wind, shallows, current, wave)
External cause, e.g., injured by another water sports participant
Defect in equipment/gear

e. How did the injury happen?
Impact on water
Impact on land
Due to rapid movement during trick/maneuver execution
Contact/collision with own equipment (kite/board/lines)
Contact/collision with another kiter/water sports participant/obstacle or foreign material (kite/board/lines)
Due to overexertion without accident

f. At what point during the session did the injury occur?
At the beginning of the session ("I had just started to get into it")
Mid-session ("I was fully in the flow")
Towards the end of the session ("I was already tired and about to leave the water")

g. What was your skill level at the time of the injury?
Beginner (kite school, supervised kiting)
Apprentice (self-taught)
Intermediate (simple jumps and tricks hooked)
Advanced (difficult/high jumps, unhooked)
Professional (competitions and/or making a living from kitesurfing)

h. How were the conditions at the time of the injury, considering your skill level?
Extremely challenging
Challenging
Normal

i. What type of water did the injury occur in?
Ocean flat water
Ocean waves
Lake
River

j. Approximately how strong was the wind when the injury occurred?
<14 knots
14-20 knots
20-30 knots
>30 knots

k. What equipment were you using at the time of the injury?
Own equipment
Borrowed equipment

l. What board were you riding at the time of the injury?
Twintip with foot straps
Twintip with boots
Directional board / Waveboard without foot straps
Directional board / Waveboard with foot straps
Foilboard without foot straps
Foilboard with foot straps
Other:

m. Did you use a safety leash at the time of the accident?
No
Yes, attached to the back of the harness
Yes, attached to the side or front of the harness

n. Did you use a board leash at the time of the accident?
No
Yes, retractable leash
Yes, coiled leash
Yes, rigid leash

o. Did you wear protective gear at the time of the accident? (Multiple choices possible)
No
Impact vest
Helmet
Earplugs
Other:

p. Did you activate the quick release system?
No
I tried to activate it, but the accident happened too quickly
Yes, before the accident
Yes, after the accident

q. Did you require medical assistance due to your injury?
No, no assistance needed
General practitioner
Outpatient hospital
Inpatient hospital stay
Intensive care unit

r. For how many days were you unable to kitesurf due to the injury?

s. For how many days were you unable to work due to the injury?

t. How long did you experience discomfort after the injury?
Days
Weeks
Months
Permanent discomfort

u. Were you able to resume your kitesurfing activities after recovery?
Yes, without limitations
Yes, but at a lower level
No, I had to quit the sport
No, I wanted to quit the sport

What type of injury did you have?

20. Did you have a head, neck, or face injury?
No (→ Question 25)
Yes

21. Did you have an eye injury?
No
Blindness
Loss of vision
Abrasion/laceration wound to the eye
Inflammation/infection, allergy/allergic reaction
Fracture (e.g., orbital fracture)
Other:

22. Did you have an ear injury?
No
Permanent deafness
Permanent hearing loss
Ruptured eardrum
Abrasion/laceration wound to the ear
Inflammation/infection, allergy/allergic reaction
Surfer's Ear
Other:

23. Did you have a head/face injury?
No
Concussion with loss of consciousness
Concussion without loss of consciousness
Facial/skull/jawbone fracture
Tooth injury
Abrasion/laceration wound to the head/face
Other:

24. Did you have a neck / vertebra injury?
No
Whiplash / cervical spine sprain
Vertebral fracture with paralysis/sensory loss
Vertebral fracture without paralysis/sensory loss
Herniated disc
Abrasion/laceration wound to the neck
Other:

25. Did you have an upper extremity (shoulder/arm/hand) injury?
No (→ Question 30)
Yes

26. Did you have a shoulder/upper arm injury?
No
Collarbone fracture
Scapula fracture

Upper arm fracture (capitulum/head of humerus fracture, humeral shaft fracture)
Acromioclavicular joint separation (Tossy/Rockwood)
Shoulder dislocation
Rotator cuff tendon tear
Abrasion/laceration wound to the shoulder/upper arm
Other:

27. Did you have an elbow injury?
No
Elbow bone fracture (olecranon fracture, radial head fracture, etc.)
Elbow dislocation
Tendon/ligament rupture (elbow)
Abrasion/laceration wound to the elbow
Other:

28. Did you have a forearm injury?
No
Bone fracture (radius/ulna)
Abrasion / Tear / Laceration on the forearm
Other:

29. Did you have a hand/finger injury?
No
Hand bone fracture (wrist, carpal bones, metacarpals)
Wrist dislocation
Finger bone fracture
Finger dislocation
Abrasion/laceration wound to the hand/fingers
Tendon/ligament injury to hand/fingers
Other:

30. Did you have a trunk (back, chest, pelvis) injury?
No (→ Question 34)
Yes

31. Did you have a back injury?
No
Vertebral fracture with paralysis/sensory loss
Vertebral fracture without paralysis/sensory loss
Herniated disc
Abrasion/laceration wound to the back
Other:

32. Did you have a chest/rib/sternum injury?
No
Rib fracture
Sternum fracture
Rib dislocation
Abrasion/laceration wound to the chest
Other:

33. Did you have a pelvic injury?
No
Pelvic bone fracture
Abrasion/laceration wound to hip/pelvis/buttock
Other:

34. Did you have a lower extremity (hip, thigh, lower leg, feet) injury?
No (→ Question 40)
Yes

35. Did you have a hip/thigh injury?
No
Hip/thigh bone fracture
Hip dislocation
Abrasion/laceration wound to thigh
Other:

36. Did you have a knee joint injury?
No
Shinbone head fracture (tibial plateau fracture)
Knee dislocation
Patella (kneecap) dislocation/fracture
Quadriceps/patellar tendon rupture
Anterior cruciate ligament tear
Posterior cruciate ligament tear
Medial collateral ligament tear
Lateral collateral ligament tear
Meniscus injury
Abrasion/laceration wound to the knee
Cartilage damage
Other:

37. Did you have a lower leg injury?
No
Shinbone fracture (except tibial plateau, see knee joint)
Fibula fracture
Tibia and fibula fracture
Achilles tendon rupture
Abrasion/laceration wound to the lower leg
Other:

38. Did you have an ankle joint injury?
No
Ankle bone fracture (ankle, talus)
Ankle ligament tear
Abrasion/laceration wound to the ankle
Cartilage damage
Other:

39. Did you have a foot/toe injury?
No
Foot bone fracture (calcaneus, tarsal bones, metatarsals)
Toe bone fracture
Abrasion/laceration wound to the foot/toes
Other:

40. Did you have internal injuries?
No
Pneumothorax
Spleen rupture
Internal bleeding
Vascular injury, *Nerve injury*
Other:

41. Did you have a polytrauma (injury to multiple body parts/systems simultaneously, requiring immediate hospitalization)?
No
Yes, life-threatening
Yes, requiring intensive care unit stay
Yes, requiring regular hospital ward stay

42. Did you have any other injuries not listed above?

Free text

43. If you'd like, please briefly describe your injury and how it happened in your own words:

Free text
